# A Case of Epistaxis as the First Sign of Acute Idiopathic Thrombocytopenic Purpura

**DOI:** 10.1155/2021/6612939

**Published:** 2021-02-06

**Authors:** Shori Tajima, Fumihiko Matsumoto, Takashi Anzai, Satoshi Hara, Yo Suzuki, Katsuhisa Ikeda

**Affiliations:** Department of Otorhinolaryngology, Juntendo University Faculty of Medicine, Tokyo 113-8421, Japan

## Abstract

Idiopathic thrombocytopenic purpura (ITP) is an acquired thrombocytopenia caused by the action of autoantibodies against platelet antigens. It is traditionally defined by a platelet count of less than 10 × 10^4^/*μ*L. Most patients with ITP are asymptomatic; however, symptoms have been confirmed in some cases. Conversely, it is very rare to find epistaxis as the first sign of ITP. We report the case of an 84-year-old man who came to the ear, nose, and throat department with severe and repeated epistaxis. We decided to keep him hospitalized as it was very difficult to stop the nasal bleeding. A full blood count showed a platelet level of only 1000/*μ*L. Hematologic results confirmed the diagnosis of ITP. The patient underwent treatment with intravenous gamma-globulin, platelet transfusions, and romiplostim with a favorable response.

## 1. Introduction

Idiopathic thrombocytopenic purpura (ITP) is a common hematologic disorder characterized by the destruction of immune-mediated platelets, and it affects nearly 1 in 10,000 people around the world [1].

Approximately 80% of ITPs are idiopathic and are thought to be related to antibodies against platelets [2]. On the other hand, roughly 20% of ITPs can present as a secondary diagnosis on top of a coexisting illness such as an infectious disease [1]. ITP has been reported to be associated with *Helicobacter pylori*, cytomegalovirus, varicella-zoster virus, hepatitis C virus, and human immunodeficiency virus [3–7].

Symptoms associated with ITP are petechiae, purpura, and mucosal bleeding, and in the most serious cases, fatal intracranial hemorrhage [1]. Generally, ITP is relieved naturally if it has no clinical symptoms such as bleeding tendency. Moreover, there are few reports about ITP which was revealed by epistaxis.

We report the case of an 84-year-old man who visited our department with severe epistaxis. After packing the nasal cavity, a full blood count showed only 1000 platelets. He was diagnosed with ITP, despite him never having reported ITP in the past.

Epistaxis is a common symptom of ITP. However, it is very rare for it to be the first sign of ITP.

## 2. Clinical Case

An 84-year-old patient was admitted to the emergency department with epistaxis in his left nostril, which had not stopped despite the patient having packed the anterior nasal cavity. His medical history included prostatic hyperplasia and dyslipidemia, and he had no history or prescription of antithrombotic drugs. Nasal bleeding was observed in the left-sided Kiesselbach's area. We used bipolar coagulation techniques to relieve the bleeding. However, he was admitted to the otorhinolaryngology department two days later, presenting an epistaxis in the right nostril. Nasal bleeding occurred at various points, including the left-sided base of the middle turbinate. We attempted to coagulate the bleeding point using bipolar coagulation techniques. However, the bleeding was not relieved completely and we decided to keep the patient hospitalized.

The bleeding was almost controlled after bilateral anterior packing of the nasal cavity with Merocel® (Medtronic Inc., Minneapolis, USA) and Surgicell® (Johnson & Johnson, Tokyo, Japan). The result of a full blood count surprisingly revealed a platelet count of only 1000/*μ*L, as shown in Table 1. Based on these results, the patient was admitted to our hospital and a hematology consultation was requested.

**Figure 1:**
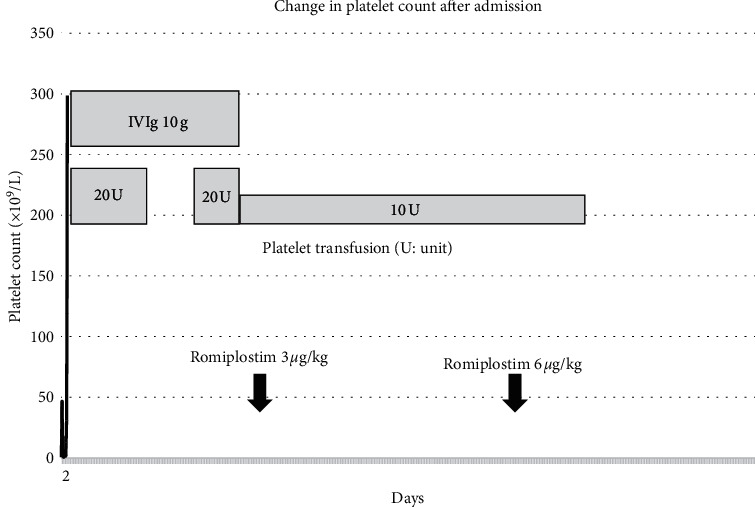
Change in platelet count after admission. The patient underwent treatment with platelet transfusion, high-dose intravenous gamma-globulin (IVIG), and romiplostim. Platelet counts increased about 14 days after admission.

The final diagnosis was ITP, and the patient underwent treatment with high-dose intravenous gamma-globulin (IVIG) and platelet transfusions. Platelet counts did not increase despite these hematologic interventions. Thus, the patient was treated with romiplostim, which is a new drug formulated for the treatment of ITP. After treatment with romiplostim, the platelet counts increased rapidly, as shown in Figure 1.

## 3. Discussion

Epistaxis is one of the most common otorhinolaryngology diseases in the emergency department. Up to 90% of all epistaxis occur within the vascular watershed area of the nasal septum, known as Kiesselbach's area [8]. To relieve anterior nosebleeds, the patient must apply proper pressure by grasping the ala nasi distally and pinching them tightly against the septum.

On the other hand, it is difficult to relieve posterior nosebleeds because the patient cannot apply pressure to the posterior nasal cavity.

In this case, the main bleeding point was found to be the left-sided base of the middle turbinate. It was necessary to coagulate the bleeding point because it was difficult for him to stop the bleeding by pressing the ala nasi. However, we could not stop the bleeding despite coagulation by bipolar coagulation techniques. In such cases, treatments such as gauze packing for severe low platelet levels should be avoided as it can lead bleeding worse and we should suspect the existence of another underlying disease such as bleeding tendency. In the present case, a full blood count showed only 1000/*μ*L platelets curiously. Thus, we decided to consult hematology for treatment of platelet disorder prior to hemostasis.

The primary treatment for ITP is corticosteroids. Approximately 80% of patients respond to corticosteroids. However, many of them relapse after tapering off steroids. It is clear that corticosteroids are an effective first-line treatment for ITP as they are considered to be safe for pregnant patients [9]. Moreover, intravenous immunoglobulin (IVIG) or Rho (D) immune globulin (anti-RhD) can be used for patients who do not respond to corticosteroids or who cannot use them due to contraindications [9].

Splenectomy was recommended as the second-line treatment after the failure of treatment with corticosteroids due to the destruction of platelets in the spleen [10]. Splenectomy has been found to be safe even in patients with a few platelets, and platelet infusion is not indicated [11]. On the other hand, splenectomy has some risks such as infection, bleeding, thrombosis, and relapse.

Moreover, rituximab, which is a monoclonal antibody against the CD20 antigen, is a new option for the treatment of ITP. However, with rituximab, there were some moderate adverse events such as infusion reactions, including life-threatening events. This suggests that it is not a safe medicine and must be used with caution. Therefore, rituximab is the last option for patients who cannot undergo splenectomy and for children with severe refractory ITP [9].

Approximately 30%–40% of adults do not respond to first-line therapy and splenectomy [12]. If these patients are at risk of severe bleeding, they are treated with prednisone alone.

Some studies on new drugs such as romiplostim which was used in the present case are currently ongoing. It is true that romiplostim helps in boosting the platelet response to levels exceeding 50 × 10^9^/L; however, there are various reports that cast doubt on whether this response is sustained [13–16]. Additionally, the side effects of romiplostim are still unknown. There are some reports of mild symptoms such as headache and upper respiratory infection, to serious events such as hepatotoxicity, arthralgias, and severe fatigue [17]. The effectiveness and side effects of romiplostim should be investigated further, and careful consideration should be given to the decision on whether or not it is suitable for treatment.

In general, epistaxis is a common symptom of ITP. However, patients with ITP usually have petechiae or purpura, which are common first symptoms of ITP [11]. However, in the present case, the patient had no symptoms other than epistaxis. We could diagnose ITP because it was strangely difficult to control the nasal bleeding.

To date, only 1 case of ITP with epistaxis as the first symptom has been reported in the published literature [18]. In this report, a 27-year-old patient was diagnosed ITP with epistaxis as the first sign of ITP. He was treated with corticosteroids and antifibrinolytics and recovered quickly. On the other hand, in the present case, an 84-year-old man experienced a more severe course. There have been no other reports of such an elderly case.

## 4. Conclusion

When controlling epistaxis is difficult despite appropriate coagulation of the bleeding point, we must be cautions and suspect the possibility of abnormal coagulation such as that seen in ITP. Moreover, in such case, platelet counts should be increased with the cooperation of hematology prior to hemostasis.

## Figures and Tables

**Table 1:** Analytical values on hospital admission.

Analytical values on hospital admission		
Biochemical markers	Blood glucose	142 mg/dL
	Potassium	3.8 mmol/L
	Sodium	144 mmol/L
	Blood urea	19 mg/dL
	Creatinine	0.85 mg/dL

Complete blood count	Leukocytes	10,300 × 106/L
	Neutrophils	8300 × 106/L
	Lymphocyte	1340 × 106/L
	Monocytes	520 × 106/L
	Haemoglobin	13.3 g/dL
	Platelets	1×109/L
