# Corrigendum to “A Case of Epistaxis as the First Sign of Acute Idiopathic Thrombocytopenic Purpura”

**DOI:** 10.1155/2021/9804515

**Published:** 2021-04-12

**Authors:** Shori Tajima, Fumihiko Matsumoto, Takashi Anzai, Satoshi Hara, Yo Suzuki, Katsuhisa Ikeda

**Affiliations:** Department of Otorhinolaryngology, Juntendo University Faculty of Medicine, Tokyo 113-8421, Japan

In the article titled “A Case of Epistaxis as the First Sign of Acute Idiopathic Thrombocytopenic Purpura” [1], Figure 1 was formatted incorrectly. The authors have corrected this error and provided the correct Figure 1 as follows:

## Figures and Tables

**Figure 1:**
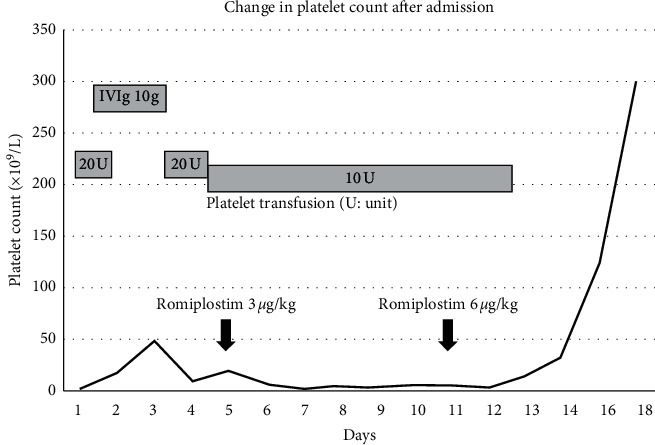
Change in platelet count after admission. The patient underwent treatment with platelet transfusion, high-dose intravenous gamma-globulin (IVIG), and romiplostim. Platelet counts increased about 14 days after admission.
